# Perspectives of patients and healthcare professionals on the impact of telemetrically supported patient self-management for chronic obstructive pulmonary disease (COPD): a qualitative study nested in the TELESCOT trial

**Published:** 2012-06-15

**Authors:** Hilary Pinnock, Peter Fairbrother, Janet Hanley, Lucy McCloughan, Allison Todd, Brian McKinstry

**Affiliations:** University of Edinburgh, UK; University of Edinburgh, UK; Edinburgh Napier University, UK; University of Edinburgh, UK; University of Edinburgh, UK; University of Edinburgh, UK

**Keywords:** telehealth, COPD, self-management, primary care, continuity of care

## Abstract

**Background:**

Early identification of exacerbations reduces hospital admissions and may slow disease progression. The TELESCOT randomised control trial based in Lothian, Scotland, is investigating the impact of a tele-monitoring service for COPD with the primary aim of reducing hospitalisation.

**Aim:**

The nested qualitative study explored the views of patients and professionals on models of telemetric service delivery and the impact on self-management.

**Method:**

We undertook semi-structured interviews with patient and professional participants at different time points in the TELESCOT COPD trial. Transcribed, coded data were analysed thematically. Interpretation was supported by multidisciplinary discussion.

**Results:**

38 patients (47% male, mean age 67.5 years) and 32 healthcare professionals provided 70 interviews. Both patients and professionals considered that home tele-monitoring had the potential to reduce the risk of hospital admission. Patients generally appreciated being ‘watched over’ by the tele-monitoring, which gave them confidence to manage their own condition. They used tele-data to improving their understanding of COPD, determine their current state of health and influence decisions about their daily activities. Numerical data (e.g. oxygen saturations) were particularly valued. Changes in readings validated their decisions to adjust treatment or seek timely professional advice, and eased access to clinical care. Patients valued the personalised care provided by tele-monitoring staff familiar with their circumstances and state of health. Professionals emphasised the potential role of telemetry in encouraging prompt compliance with medically defined behaviours and attitudes, though some doubted whether it would be sufficient to overcome a perceived reluctance on the part of patients to acknowledge and take ownership of the disease. There was also a concern that ‘fixation’ on monitoring physiological parameters (especially oxygen saturation levels), promoted a medical model of the disease and might increase dependence on services in some patients. The GPs and community nursing or physiotherapy teams who provided the supporting services emphasised the importance of ‘knowing the patient’ and ‘knowing what’s normal for the individual’ in using their clinical skills to interpret incoming tele-monitoring data.

**Conclusion:**

Enthusiasm for tele-monitoring as a means of facilitating self-management and thereby reducing admissions is tempered by concerns about increased medicalisation and dependence on support services. Tele-monitoring provides data which can be used to support self-management decisions and acts as a channel for seeking professional support. The patient-practitioner relationship, personalisation and continuity of care were prioritised as important elements in delivering clinical support for tele-monitoring services by patients and professionals.

